# Associations of Dietary Vitamin A and Beta-Carotene Intake With Depression. A Meta-Analysis of Observational Studies

**DOI:** 10.3389/fnut.2022.881139

**Published:** 2022-04-25

**Authors:** Yi Zhang, Jun Ding, Jieyu Liang

**Affiliations:** ^1^Department of Orthopaedics, Xiangya Hospital, Central South University, Changsha, China; ^2^National Clinical Research Center for Geriatric Disorders, Xiangya Hospital, Central South University, Changsha, China; ^3^Changsha Social Work College, Changsha, China

**Keywords:** dietary vitamin A intake, dietary beta-carotene intake, depression, meta-analysis, observational studies

## Abstract

**Objective:**

To clarify the associations of dietary vitamin A and beta-carotene intake with depression based on a meta-analysis of observational studies.

**Methods:**

An extensive literature search on February 2022 (PubMed, Web of Science and Embase) was employed to identify observational studies on the associations of dietary vitamin A and beta-carotene intake with depression. The pooled relative risk (RR) of depression for the highest vs. lowest dietary vitamin A and beta-carotene intake category, and the standard mean difference (SMD) of dietary vitamin A and beta-carotene intake for depression vs. control subjects, were calculated.

**Results:**

A total of 25 observational studies (100,955 participants), which included 24 cross-sectional/case-control and 1 prospective cohort study, were included in this study. The overall multi-variable adjusted RR demonstrated that dietary vitamin A intake was inversely associated with depression (*RR* = 0.83, 95%CI: 0.70–1.00; *P* = 0.05). In addition, the combined SMD showed that the dietary vitamin A intake in depression was also lower than that in control subjects (SMD = −0.13, 95%CI: −0.18 to −0.07; *P* < 0.001). On the other hand, the overall multi-variable adjusted RR indicated that dietary beta-carotene intake was negatively associated with depression (*RR* = 0.63, 95%CI: 0.55–0.72; *P* < 0.001). The combined SMD showed that the dietary beta-carotene intake in depression was also lower than that in control subjects (SMD = −0.34, 95%CI: −0.48 to −0.20; *P* < 0.001).

**Conclusion:**

Our results suggest that both dietary vitamin A and beta-carotene intake is inversely associated with depression. However, due to the limited evidence, further prospective cohort studies are still needed.

## Introduction

Depression, one of the most common global mental disorders, affects females twice as much as males (1). The usual symptoms of depression are exhaustion, sadness, lack of interest in daily activities and suicide (2). Depression has affected approximately 300 million people (3), and is estimated to be the leading cause of disability worldwide by 2030 (4). Most importantly, low- and middle-income countries (LMICs) may be disproportionally suffered from depression. More than 80% of global disability due to depression comes from LMICs, and the majority of subjects suffered from depression in LMICs do not receive appropriate treatment (5). Since emerging evidence has indicated the significant role of dietary factors in depression (6, 7), the identification of affordable and accessible dietary factors for depression is important in its clinical management, especially in LMICs.

Vitamin A, a generic term for compounds with retinol biological activity, is usually found in foods derived from animal products (8, 9). Generally speaking, vitamin A is related to several physiological processes, such as differentiation and function of immune system, embryo development, vision, and energy metabolism (10). On the other hand, synthesized by photosynthetic organisms, carotenoids are served as light-harvesting scavengers during photosynthesis. Beta-carotene, the most common carotene in nature (10), is served as an important vitamin A precursor. On the contrary to vitamin A, beta-carotene is mainly derived from plant products (8). As a natural antioxidant, carotenoids protect organisms from oxidative damage *via* removing reactive oxygen species (ROS) and other free radicals (11). Interestingly, fundamental evidence has indicated the antidepressant property of beta-carotene, which may be associated with the reduced levels of tumor necrosis factor-a (TNF-α) and interleukin-6 (IL-6), and increased levels of brain-derived neurotrophic factor (BDNF) (12). Therefore, it seems naturally that dietary vitamin A and beta-carotene intake is negatively associated with depression.

To our best knowledge, a number of observational studies have investigated the associations of dietary vitamin A and beta-carotene intake with depression (13–37). However, no final conclusion is obtained. The present meta-analysis is therefore employed to clarify the issue. It is hypothesized that both dietary vitamin A and beta-carotene intake is inversely associated with depression.

## Materials and Methods

### Search Strategy

This meta-analysis study was employed according with the Preferred Reporting Items for Systematic Reviews and Meta-analyses (PRISMA) guidelines (38). The electronic database of PubMed, Web of Science and Embase were searched through during February 2022 (no restriction was set for the initiate time) using a combination of keywords and in-text words related to depression (“depression,” “depressive”), vitamin A (“vitamin A,” “retinol,” “prepalin”) and beta-carotene (“carotene,” “carotin,” “carotenoid”). No language restrictions were imposed in the search. To identify eligible studies, the titles and abstracts of all articles were first screened. Then, the full articles were read to include the eligible studies. Moreover, the references of the retrieved articles and reviews were also evaluated.

### Study Selection

Two researchers reviewed the titles, abstracts and full texts of the retrieved studies independently for relevance evaluation, and disagreements (if any) were resolved by discussions. The included studies were required to meet the following criteria: (1) observational studies; (2) the associations of dietary vitamin A and beta-carotene intake with depression; (3) odds ratio (OR), relative risk (RR) or standard mean difference (SMD) reported. The exclusion criteria were listed as follows: (1) duplicated or irrelevant articles; (2) reviews, letters or case reports; (3) randomized controlled trials; and (4) non-human studies.

### Data Extraction

The quality of each included study was evaluated in accordance with the Newcastle-Ottawa (NOS) criteria for non-randomized studies. It contains 8 items categorized into three dimensions: (1) the selection of study groups; (2) the comparability among different groups; (3) the identification of exposure or outcome of study cohorts, respectively. The included cross-sectional/case-control studies were assessed by using NOS for case-control studies, whereas cohort studies were assessed by using NOS for cohort studies. Disagreements (if any) were resolved through discussions until a consensus was reached.

The extracted data included the first author, year of publication, location, age, sex, sample size, study design, adjustments, exposure assessment, category of exposure, effect estimates, and diagnostic criteria of depression. The corresponding effect estimates with 95% CIs for the highest vs. lowest dietary vitamin A and carotene intake category were extracted (adjusted for the maximum number of confounding variables). Moreover, the dietary vitamin A and beta-carotene intake (mean ± SD) was also extracted for depression vs. control subjects to calculate the SMD.

### Statistical Analyses

The RR for depression and SMD for dietary vitamin A and beta-carotene intake were the outcome measures in our study. The *I*^2^ statistic was examined to measure the percentage of total variation across studies due to heterogeneity (*I*^2^ > 50% was considered as heterogeneity). The random-effects model was accepted if significant heterogeneity was observed among the studies; otherwise, the fixed effects model was utilized. The publication bias was assessed by Begg’s test (39). A *p*-value < 0.05 was considered as statistically significant. Moreover, subgroup analysis was employed for geographical region, exposure assessment, sex, population, sample size, study design, and adjustment of BMI and energy intake.

## Results

### Study Identification and Selection

**FIGURE 1:**
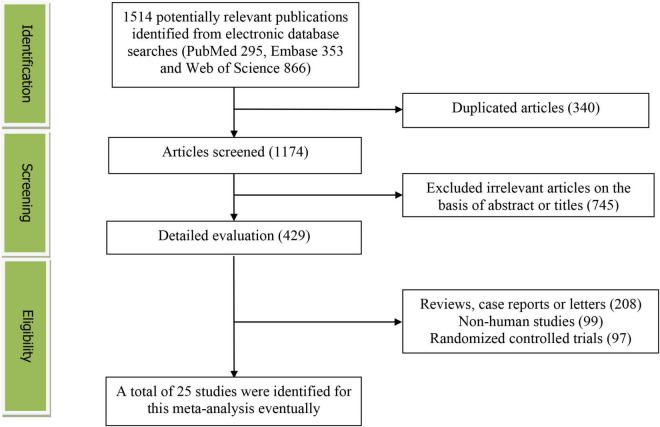
The detailed flow diagram of the study identification and selection in this meta-analysis.

Figure 1 presents the study screening process. During the initial literature search, a total of 1,514 potentially relevant articles (295 for PubMed, 353 for Embase and 866 for Web of Science) were retrieved. After eliminating 340 duplicated articles, 1,174 articles were screened according to the titles and abstracts. 745 irrelevant studies were removed. Then, 208 reviews, case reports or letters, 99 non-human studies and 97 randomized control trials studies were excluded. Eventually, 25 studies (24 cross-sectional/case-control and 1 prospective cohort study) were selected for this meta-analysis (13–37).

### Study Characteristics

The characteristics and NOS score of all the included studies are shown in Table 1. These studies were published between 2009 and 2022. 14 of the included studies were performed in Asian countries [Korea (14, 20, 21, 25, 29, 30, 35, 36), Iran (18, 31, 33), Japan (13, 23) and China (28)], and the other ones were conducted in United States (15, 17, 27, 32, 34), Brazil (26, 37), Australia (16), Spain (22, 24), and Turkey (19). Male, female and both male and female participants were recruited in 1 (18), 8 (14, 20, 26, 28, 30, 31, 33, 36), and 16 (13, 15–17, 19, 21–25, 27, 29, 32, 34, 35, 37) studies, respectively. The sample size ranged from 41 to 17,401 for a total number of 100,955. The exposure was assessed by food-frequency questionnaire (FFQ) in 16 studies (13–16, 18, 20, 21, 23–25, 28, 29, 31, 33, 36, 37), and recall method in 9 studies (17, 19, 22, 26, 27, 30, 32, 34, 35). The diagnostic criteria of depression were Diagnostic and Statistical Manual of Mental Disorders-IV (DSM-IV) (15, 17, 19, 24), Patient Health Questionnaire-9 (PHQ-9) (27, 29, 32, 34), Center for Epidemiological Studies Depression Scale (CES-D) (13, 14, 16, 22, 28, 30), Beck Depression Inventory (BDI) (18, 20, 21, 26, 33, 36), Geriatric Depression Scale (GDS) (23), Clinical Interview Schedule Revised (CIS-R) (37), and Depression, Anxiety, Stress Scale (DASS) (31), respectively.

**TABLE 1:** Characteristics of the individual studies included in this meta-analysis.

References	Location	Age years	Sex	Sample size	Study design	Adjustments	Exposure	Category of exposure	Effect estimates	Diagnostic criteria of depression	NOS
Oishi et al. (13)	Japan	65–75	Both	401	Cross-sectional	Age, chronic diseases, BMI and social support	FFQ	Male Vitamin A Tertile 1 Tertile 2 Tertile 3 Beta-carotene Tertile 1 Tertile 2 Tertile 3 Female Vitamin A Tertile 1 Tertile 2 Tertile 3 Beta-carotene Tertile 1 Tertile 2 Tertile 3	1.00 0.67 (0.23, 1.70) 0.78 (0.28, 2.17) 1.00 0.52 (0.19, 1.45) 0.36 (0.13, 0.98) 1.00 0.52 (0.20, 1.37) 1.00 (0.39, 2.58) 1.00 0.55 (0.21, 1.40) 0.52 (0.20, 1.35)	CES-D	8
Park et al. (14)	Korea	20	Female	130	Case-control	NA	FFQ	Control Depression Control Depression	Vitamin A 641.3 (587.4, 695.2) 539.5 (484.4, 594.6) Beta-carotene 2656.0 (2394.1, 2917.9) 2076.6 (1773.9, 2379.3)	CES-D	7
Payne et al. (15)	United States	>60	Both	278	Case-control	NA	FFQ	Control Depression	Beta-carotene 4136.7 (3663.0, 4610.4) 3759.0 (3275.1, 4242.9)	DSM-IV	7
Purnomo et al. (16)	Australia	>18	Both	58	Case-control	NA	FFQ	Control Depression	Vitamin A 1524.8 (925.6, 2124.0) 801.5 (593.1, 1009.9)	CES-D	5
Beydoun et al. (17)	United States	20–85	Both	1,798	Cross-sectional	NA	Recall method	Male Control Depression Female Control Depression	Beta-carotene 2190.2 (1875.6, 2504.8) 1535.7 (1223.1, 1848.3) Beta-carotene 2364.5 (2127.6, 2601.4) 1368.4 (857.3, 1879.5)	DSM-IV	8
Prohan et al. (18)	Iran	18–25	Male	60	Case-control	NA	FFQ	Control Depression	Beta-carotene 2890.6 (2713.0, 3068.2) 2425.1 (2223.8, 2626.4)	BDI	6
Kaner et al. (19)	Turkey	18–60	Both	59	Case-control	NA	Recall method	Control Depression	Vitamin A 670.5 (449.6, 1249.6) 516.6 (467.7, 683.5)	DSM-IV	6
Kim et al. (20)	Korea	12–18	Female	849	Case-control	Energy intake and menstrual regularity	FFQ	Vitamin A Tertile 1 Tertile 2 Tertile 3 Beta-carotene Tertile 1 Tertile 2 Tertile 3	1.00 0.73 (0.43, 1.22) 0.60 (0.32, 1.13) 1.00 0.79 (0.48, 1.31) 0.54 (0.29, 0.99)	BDI	7
Jeong et al. (21)	Korea	20–65	Both	734	Cross-sectional	NA	FFQ	Male Control Depression Female Control Depression	Vitamin A 1177.0 (476.2, 1877.8) 792.8 (536.4, 1049.2) Vitamin A 820.4 (752.8, 888.0) 829.9 (619.7, 1040.1)	BDI	7
Rubio-López et al. (22)	Spain	6–9	Both	710	Cross-sectional	NA	Recall method	Control Depression	Vitamin A 481.9 (472.8, 491.0) 461.7 (442.7, 480.7)	CES-D	7
Nguyen et al. (23)	Japan	>65	Both	1,634	Cross-sectional	NA	FFQ	Control Depression Control Depression	Vitamin A 401.3 (384.6, 418.0) 361.6 (342.5, 380.7) Beta-carotene 2176.6 (2096.5, 2256.7) 1158.1 (1029.5, 1286.7)	GDS	7
Sánchez-Villegas et al. (24)	Spain	38	Both	13,983	Cohort	Sex, age, physical activity, BMI, energy intake, special diets, smoking, alcohol intake and prevalence of CVD, HTA or T2DM	FFQ	Vitamin A Inadequacy Adequacy	1.00 0.72 (0.53, 0.98)	DSM-IV	9
Seo and Je (25)	Korea	19–64	Both	10,591	Cross-sectional	Age, survey year, total energy intake, BMI, marital status and physical activity	FFQ	Male Control Depression Male Control Depression Female Control Depression	Vitamin A 583.2 (581.2, 585.2) 563.2 (561.1, 565.3) Beta-carotene 2852.6 (2850.6, 2854.6) 2724.7 (2722.6, 2726.8) Vitamin A 563.6 (561.6, 565.6) 560.4 (558.3, 562.5)	Physician diagnosis	7
								Female Control Depression	Beta-carotene 2777.2 (2096.5, 2256.7) 2761.7 (2759.6, 2793.8)		
de Oliveira et al. (26)	Brazil	50–69	Female	41	Case-control	NA	Recall method	Control Depression	Vitamin A 878.5 (498.2, 1258.8) 515.9 (285.7, 746.1)	BDI	5
Iranpour and Sabour (27)	United States	> 18	Both	4,737	Cross-sectional	NA	Recall method	Control Depression Control Depression	Vitamin A 628.8 (606.5, 651.1) 470.0 (393.3, 546.7) Beta-carotene 1905.5 (1804.1, 2006.9) 1233.6 (969.1, 1498.1)	PHQ-9	8
Li and Li (28)	China	42–52	Female	2,762	Cross-sectional	Age, race/ethnicity, total family income, sex hormone binding globulin, DBP, BMI, TG, LDLC, HDLC, SHBG, Dietary caloric intake and Dietary Fat intake	FFQ	Control Depression Beta-carotene Quartile 1 Quartile 2 Quartile 3 Quartile 4	Beta-carotene 2.07 (1.33, 3.38) 1.83 (1.18, 3.03) 1.00 0.88 (0.69, 1.12) 0.76 (0.59, 0.97) 0.74 (0.57, 0.94)	CES-D	7
Park et al. (29)	Korea	20–60	Both	5,897	Cross-sectional	NA	FFQ	Male Control Depression Male Control Depression	Vitamin A 839.7 (791.4, 888.0) 820.8 (720.3, 921.3) Beta-carotene 4117.3 (3894.8, 4399.8) 4185.9 (3635.4, 4736.4)	PHQ-9	7
								Female Control Depression Female Control Depression	Vitamin A 740.5 (698.0, 783.0) 616.7 (559.6, 673.8) Beta-carotene 3771.7 (3526.4, 4017.0) 3184.2 (2869.1, 3499.3)		
Park et al. (30)	Korea	22	Female	178	Cross-sectional	NA	Recall method	Control Depression	Vitamin A 552.5 (493.7, 611.3) 447.9 (383.0, 512.8)	CES-D	7
Farhadnejad et al. (31)	Iran	15–18	Female	263	Cross-sectional	Age, BMI, physical activity, mother/father’s education level, dietary fiber, and total energy intake	FFQ	Control Depression Beta-carotene Tertile 1 Tertile 2 Tertile 3	Beta-carotene 4460.0 (4013.6, 4906.4) 4305.0 (3589.7, 5020.3) 1.00 0.48 (0.25, 0.90) 0.46 (0.23, 0.95)	DASS	7
Ge et al. (32)	United States	18–80	Both	17,401	Cross-sectional	Age and gender, ethnicity, educational level, BMI, annual family income, work activity, recreational activity, hypertension, diabetes, smoking status, drinking status, and total energy intake	Recall method	Control Depression Beta-carotene Quartile 1 Quartile 2 Quartile 3 Quartile 4	Beta-carotene 1112.5 (1076.3, 1148.7) 669.0 (592.6, 745.4) 1.00 0.65 (0.51, 0.83) 0.54 (0.42, 0.70) 0.59 (0.47, 0.75)	PHQ-9	8
Khayyatzadeh et al. (33)	Iran	12–18	Female	988	Cross-sectional	Age, energy intake, menstruation, family members, parental death, parental divorce, physical activity and BMI	FFQ	Control Depression Control Depression Beta-carotene Quartile 1 Quartile 2 Quartile 3 Quartile 4	Vitamin A 600.7 (574.3, 627.1) 584.6 (487.1, 682.1) Beta-carotene 3558.0 (3349.5, 3766.5) 3024.0 (2737.8, 3310.2) 1.00 0.91 (0.58, 1.42) 0.77 (0.50, 1.20) 0.42 (0.26, 0.69)	BDI	7
Lin and Shen (34)	United States	> 18	Both	4,105	Cross-sectional	Age, gender, marital status, race, educational level, body mass index, smoke, alcohol drinking, family income, work activity, recreational activity, hypertension, hypercholesterolemia, diabetes, total daily energy intake, zinc intake, selenium intake, magnesium intake, total polyunsaturated fat intake, vitamin B6 intake, vitamin B12 intake and folate intake	Recall method	Control Depression Beta-carotene Tertile 1 Tertile 2 Tertile 3	Beta-carotene 1060.5 (991.0, 1130.0) 625.0 (451.6, 798.4) 1.00 0.77 (0.47, 1.26) 0.81 (0.48, 1.38)	PHQ-9	8
Nguyen et al. (35)	Korea	> 10	Both	16,371	Cross-sectional	NA	Recall method	Control Depression	Vitamin A 481.4 (473.7, 489.3) 392.8 (358.8, 430.0)	Physician diagnosis	8
Park et al. (36)	Korea	45–69	Female	2,190	Cross-sectional	Age, BMI, education level, household income, marital status, job, current alcohol drinking, current smoking, physical activity, chronic disease status, sleep duration, family history of depression, stress, menopause status, and total energy intake	FFQ	Control Depression Beta-carotene Quartile 1 Quartile 2 Quartile 3 Quartile 4	Beta-carotene 6.5 (6.3, 6.6) 6.0 (5.7, 6.2) 1.00 0.82 (0.59, 1.12) 0.90 (0.64, 1.27) 0.82 (0.55, 1.22)	BDI	8
Ferriani et al. (37)	Brazil	35–74	Both	14,737	Cross-sectional	Total calorie, age, race, total cholesterol, HDL cholesterol, systolic blood pressure, antihypertensive drug, diabetes, and smoking, cardiovascular disease and physical activity	FFQ	Control Depression Vitamin A Quintile 1 Quintile 2 Quintile 3 Quintile 4 Quintile 5	Vitamin A 910.7 (695.9, 1125.5) 877.9 (684.4, 1071.4) 1.00 1.03 (0.81, 1.32) 0.86 (0.67, 1.12) 0.86 (0.66, 1.12) 0.97 (0.75, 1.26)	CIS-R	9

### Relative Risk of Depression for the Highest vs. Lowest Category of Dietary Vitamin A Intake

**FIGURE 2:**
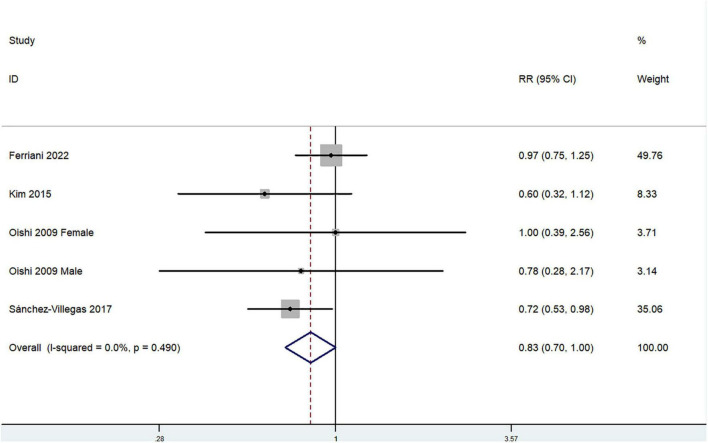
Forest plot of meta-analysis: Overall multi-variable adjusted RR of depression for the highest vs. lowest category of dietary vitamin A intake.

The overall multi-variable adjusted RR demonstrated that dietary vitamin A intake was negatively associated with depression (*RR* = 0.83, 95%CI: 0.70–1.00; *P* = 0.05) (Figure 2). No substantial level of heterogeneity was observed among various studies (*P* = 0.49, *I*^2^ = 0%). No evidence of publication bias existed according to the Begg’s rank-correlation test (*P* = 1.00). Table 2 presents the results of subgroup analysis. Such results only existed in females (*RR* = 0.75, 95%CI: 0.58–0.98; *P* = 0.03), cohort (*RR* = 0.72, 95%CI: 0.53–0.98) and adjustment of BMI (*RR* = 0.75, 95%CI: 0.56–0.99; *P* = 0.04) and energy intake (*RR* = 0.70, 95%CI: 0.53–0.92; *P* = 0.01) studies.

**TABLE 2:** Subgroup analysis of depression for the highest vs. lowest category of dietary vitamin A intake.

Stratification	Number of studies	Pooled RR	95% CI	*P*-value	Heterogeneity
All studies	4	0.83	0.70, 1.00	*P* = 0.05	*P* = 0.49; *I*^2^ = 0%
Sex					
Male	2	0.77	0.48, 1.24	*P* = 0.29	*P* = 0.98; *I*^2^ = 0%
Female	3	0.75	0.58, 0.98	*P* = 0.03	*P* = 0.65; *I*^2^ = 0%
Study design					
Cross-sectional	3	0.90	0.72, 1.13	*P* = 0.38	*P* = 0.56; *I*^2^ = 0%
Cohort	1	0.72	0.53, 0.98	/	/
Adjustment of BMI					
Adjusted	2	0.75	0.56, 0.99	*P* = 0.04	*P* = 0.81; *I*^2^ = 0%
Unadjusted	2	0.91	0.71, 1.15	*P* = 0.41	*P* = 0.17; *I*^2^ = 48%
Adjustment of energy intake					
Adjusted	2	0.70	0.53, 0.92	*P* = 0.01	*P* = 0.61; *I*^2^ = 0%
Unadjusted	2	0.96	0.75, 1.22	*P* = 0.74	*P* = 0.92; *I*^2^ = 0%

### Standard Mean Difference of Dietary Vitamin A Intake for Depression vs. Control Subjects

**FIGURE 3:**
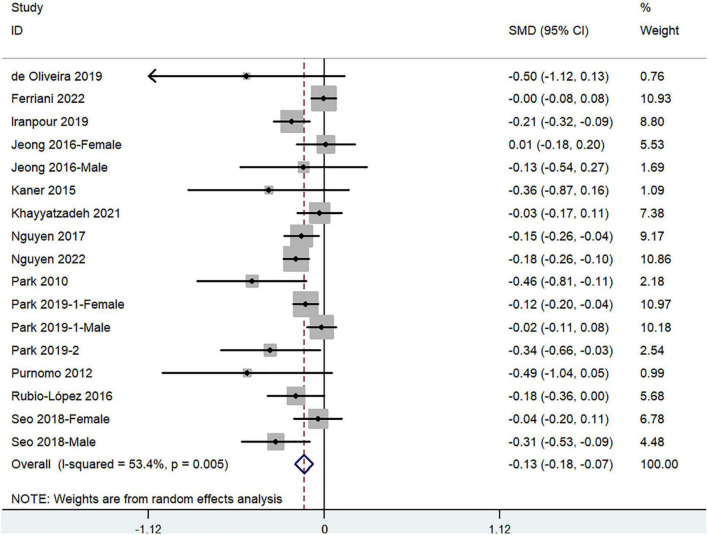
Forest plot of meta-analysis: SMD of dietary vitamin A intake for depression vs. control subjects.

The overall combined SMD showed that dietary vitamin A intake in depression was lower than that in control subjects (SMD = −0.13, 95%CI: −0.18 to −0.07; *P* < 0.001) (Figure 3). A substantial level of heterogeneity was observed among the various studies (*P* = 0.005, *I*^2^ = 53.4%). No evidence of publication bias existed according to the Begg’s rank-correlation test (*P* = 0.149). Table 3 presents the results of subgroup analysis.

**TABLE 3:** Subgroup analysis for SMD of dietary vitamin A intake in depression vs. control subjects.

Stratification	Number of studies	Pooled SMD	95% CI	*P*-value	Heterogeneity
All studies	14	−0.13	−0.18, −0.07	*P* < 0.001	*P* = 0.005; *I*^2^ = 53%
Geographical region					
Asia	7	−0.13	−0.20, −0.06	*P* < 0.001	*P* = 0.03; *I*^2^ = 51%
Non−Asia	7	−0.14	−0.25, −0.02	*P* = 0.02	*P* = 0.02; *I*^2^ = 59%
Exposure assessment					
FFQ	8	−0.07	−0.11, −0.03	*P* < 0.001	*P* = 0.03; *I*^2^ = 50%
Recall method	6	−0.20	−0.26, −0.14	*P* < 0.001	*P* = 0.82; *I*^2^ = 0%
Sex					
Male	5	−0.06	−0.12, 0.01	*P* = 0.10	*P* = 0.16; *I*^2^ = 38%
Female	9	−0.11	−0.20, −0.03	*P* = 0.007	*P* = 0.02; *I*^2^ = 56%
Population					
Adolescent	4	−0.20	−0.38, −0.03	*P* = 0.02	*P* = 0.07; *I*^2^ = 58%
Middle aged and elderly	12	−0.12	−0.18, −0.06	*P* < 0.001	*P* = 0.01; *I*^2^ = 54%
Sample size					
<1,000	8	−0.13	−0.22, −0.05	*P* = 0.002	*P* = 0.11; *I*^2^ = 39%
>1,000	6	−0.11	−0.18, −0.05	*P* < 0.001	*P* = 0.004; *I*^2^ = 66%

### Relative Risk of Depression for the Highest vs. Lowest Category of Dietary Beta-Carotene Intake

**FIGURE 4:**
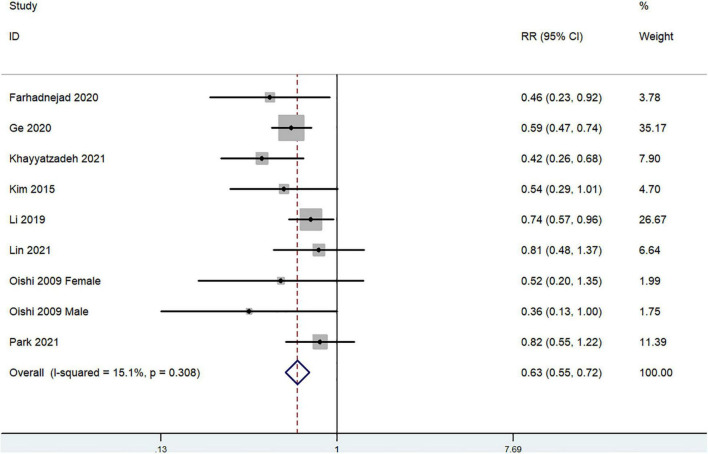
Forest plot of meta-analysis: Overall multi-variable adjusted RR of depression for the highest vs. lowest category of dietary beta-carotene intake.

The overall multi-variable adjusted RR demonstrated that dietary beta-carotene intake was negatively associated with depression (*RR* = 0.63, 95%CI: 0.55–0.72; *P* < 0.001) (Figure 4). No substantial level of heterogeneity was observed among various studies (*P* = 0.308, *I*^2^ = 15.1%). No evidence of publication bias existed according to the Begg’s rank-correlation test (*P* = 0.251). Table 4 presents the results of subgroup analysis. Such results only existed in adjustment of BMI (*RR* = 0.62, 95%CI: 0.54–0.72; *P* < 0.001) studies.

**TABLE 4:** Subgroup analysis of depression for the highest vs. lowest category of dietary beta-carotene intake.

Stratification	Number of studies	Pooled RR	95% CI	*P*-value	Heterogeneity
All studies	8	0.63	0.55, 0.72	*P* < 0.001	*P* = 0.31; *I*^2^ = 15%
Geographical region					
Asia	6	0.64	0.54, 0.76	*P* < 0.001	*P* = 0.22; *I*^2^ = 27%
Non-Asia	2	0.62	0.50, 0.76	*P* < 0.001	*P* = 0.28; *I*^2^ = 16%
Exposure assessment					
FFQ	6	0.64	0.54, 0.76	*P* < 0.001	*P* = 0.22; *I*^2^ = 27%
Recall method	2	0.62	0.50, 0.76	*P* < 0.001	*P* = 0.28; *I*^2^ = 16%
Sex					
Male	1	0.36	0.13, 1.00	/	/
Female	6	0.65	0.54, 0.78	*P* < 0.001	*P* = 0.23; *I*^2^ = 28%
Population					
Adolescent	3	0.46	0.33, 0.64	*P* < 0.001	*P* = 0.82; *I*^2^ = 0%
Middle aged and elderly	4	0.67	0.58, 0.78	*P* < 0.001	*P* = 0.42; *I*^2^ = 0%
Sample size					
< 1,000	4	0.46	0.34, 0.62	*P* < 0.001	*P* = 0.95; *I*^2^ = 0%
> 1,000	4	0.68	0.59, 0.80	*P* < 0.001	*P* = 0.37; *I*^2^ = 5%
Adjustment of BMI					
Adjusted	6	0.62	0.54, 0.72	*P* < 0.001	*P* = 0.22; *I*^2^ = 28%
Unadjusted	2	0.68	0.46, 1.02	*P* = 0.06	*P* = 0.33; *I*^2^ = 0%
Adjustment of energy intake					
Adjusted	5	0.61	0.52, 0.71	*P* < 0.001	*P* = 0.27; *I*^2^ = 22%
Unadjusted	3	0.69	0.54, 0.89	*P* = 0.003	*P* = 0.34; *I*^2^ = 8%

### Standard Mean Difference of Dietary Beta-Carotene Intake for Depression vs. Control Subjects

**FIGURE 5:**
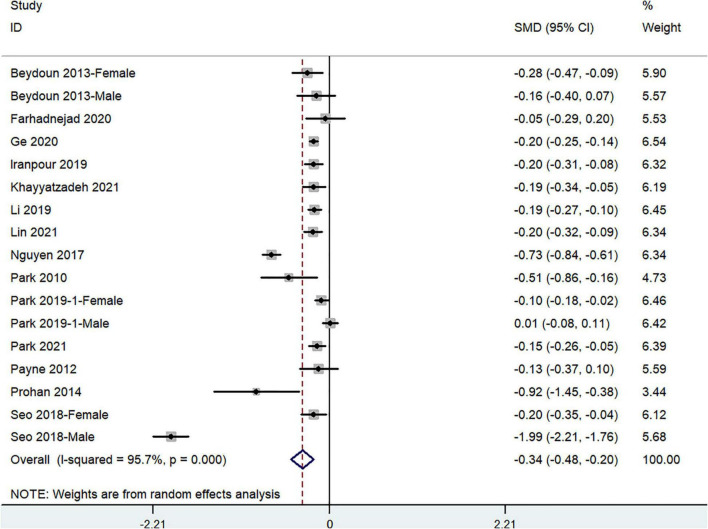
Forest plot of meta-analysis: SMD of dietary beta-carotene intake for depression vs. control subjects.

The overall combined SMD showed that dietary beta-carotene intake in depression was lower than that in control subjects (SMD = −0.34, 95%CI: −0.48 to −0.20; *P* < 0.001) (Figure 5). A substantial level of heterogeneity was observed among the various studies (*P* < 0.001, *I*^2^ = 95.7%). A publication bias existed according to the Begg’s rank-correlation test (*P* = 0.044). Table 5 presents the results of subgroup analysis. Such results only existed in females (SMD = −0.16, 95%CI: −0.12 to −0.20; *P* < 0.001) study.

**TABLE 5:** Subgroup analysis for SMD of dietary beta-carotene in depression vs. control subjects.

Stratification	Number of studies	Pooled SMD	95% CI	*P*-value	Heterogeneity
All studies	14	−0.34	−0.48, −0.20	*P* < 0.001	*P* < 0.001; *I*^2^ = 96%
Geographical region					
Asia	9	−0.43	−0.67, −0.20	*P* < 0.001	*P* < 0.001; *I*^2^ = 97%
Non−Asia	6	−0.20	−0.24, −0.16	*P* < 0.001	*P* = 0.96; *I*^2^ = 0%
Exposure assessment					
FFQ	10	−0.41	−0.63, −0.18	*P* < 0.001	*P* < 0.001; *I*^2^ = 97%
Recall method	4	−0.20	−0.24, −0.16	*P* < 0.001	*P* = 0.95; *I*^2^ = 0%
Sex					
Male	5	−0.63	−1.32, 0.07	*P* = 0.08	*P* < 0.001; *I*^2^ = 99%
Female	9	−0.16	−0.20, −0.12	*P* < 0.001	*P* = 0.33; *I*^2^ = 13%
Population					
Adolescent	4	−0.33	−0.59, −0.06	*P* = 0.02	*P* = 0.01; *I*^2^ = 73%
Middle aged and elderly	10	−0.34	−0.50, −0.17	*P* < 0.001	*P* < 0.001; *I*^2^ = 97%
Sample size					
<1,000	5	−0.27	−0.47, −0.07	*P* = 0.008	*P* = 0.02; *I*^2^ = 65%
>1,000	9	−0.35	−0.52, −0.18	*P* < 0.001	*P* < 0.001; *I*^2^ = 97%

## Discussion

A total of 25 observational studies were included in the present meta-analysis. The pooled analysis showed that both dietary vitamin A and beta-carotene intake was inversely associated with depression.

The negative associations of dietary vitamin A and beta-carotene intake with depression can be explained as follow. First, oxidative stress plays a significant role in the pathophysiology of depression (40, 41). Equipped with extended π-electron system, carotenoids stabilize unpaired electrons after radical quenching. As potent scavengers for singlet oxygen and peroxyl radicals, carotenoids act through hydrogen acceptance/abstraction, donation, electron acceptance, or physical quenching (42, 43). Second, the levels of IL-6 and TNF-α are significantly increased in depression, which impairs the expression of BDNFs and then contributes to depression (44). Beta-carotene may lead to a reduction in levels of IL-6 and TNF-α mRNA *in vivo* (12). Third, carotenoids may act through indirect pathways and cellular signaling cascades, such as nuclear factor κB (NF-κB), mitogen-activated protein kinase (MAPK) and nuclear factor erythroid 2-related factor 2 (Nrf2) (45, 46), which are closely associated with the pathology of depression (47–50). On the other hand, randomized controlled trials have indicated the potential therapeutic effect of vitamin A supplementation on depression (51), and the dietary pattern rich in vitamin A may also exert beneficial effect on depression (52–54). Taken together, current fundamental and clinical evidence is consistence with our results.

Interestingly, some of our findings are only obtained in females [the females may be more precise and reliable in the exposure assessment (55)], it may be attributed to the potential genetic sexual differences in diet-related pathology of depression (56, 57). Importantly, the inverse relationship between dietary vitamin A intake and depression only exists in prospective cohort study, but not cross-sectional study. Although the number of prospective cohort studies is rather limited (only 1), the factors that matter the dietary vitamin A and beta-carotene intake may change after depression. For instance, depressive subjects may consume less dietary vitamin A and beta-carotene due to the reduced appetite (reversed causality). Moreover, the result of subgroup analysis suggests that BMI and energy intake may also influence the overall result. Taken together, more well-designed prospective cohort studies with sexual specification are still needed.

Since vitamin A and beta-carotene are affordable and accessible nutritional factors, our findings may build an awareness with the potential collaboration between physicians and nutritionists (especially in LMICs). Nevertheless, the safety issue should also be emphasized. For instance, excessive carotenoid intake may lead to orange/yellowish skin coloration (carotenoderma or carotenemia) (58). Moreover, long-term intake of vitamin A for several months can lead to a chronic toxicity (10 mg/day for adults and 7.5–15 mg/day in children) (59). In addition, acute vitamin A toxicity cannot be ignored neither (more than 500 mg/day in adults, and 100 mg/day in children or 30 mg/day in infants) (58). The main symptoms include irritability, nausea, blurry vision, vomiting, reduced appetite, hair loss, headaches, papilledema, hemorrhage, muscle pain, weakness, altered mental status, and drowsiness (60, 61). Therefore, a careful validation for its clinical application is still needed.

Several strengthens in our study should be emphasized. First, this is the first meta-analysis study on the associations of dietary vitamin A and beta-carotene intake with depression based on observational studies. Moreover, our findings may encourage to build the potential collaboration between physicians and nutritionists for depression management (especially in LMICs). Our study is also restricted to the following issues. First, due to the limited evidence, only 1 prospective cohort studies were identified (precludes causal relationships). Second, our results may be influenced by the substantial level of heterogeneity. Third, the classification of exposure and diagnostic criteria of depression varies greatly among individuals. Forth, the adjusted factors were not uniform. Fifth, the circulating level of vitamin A and beta-carotene is not considered due to the limited evidence. The significance of our study may be weakened by these limitations.

## Conclusion

Our results suggest that both dietary vitamin A and beta-carotene intake is inversely associated with depression. However, due to the limited evidence, further well-designed prospective cohort studies with sexual specification are still needed.

## Data Availability Statement

The original contributions presented in the study are included in the article/supplementary material, further inquiries can be directed to the corresponding author/s.

## Author Contributions

YZ and JL conceived the idea and drafted this manuscript and guarantor of the overall content. JD and JL selected and retrieved relevant manuscript, and assessed each study. All authors revised and approved the final manuscript.

## Conflict of Interest

The authors declare that the research was conducted in the absence of any commercial or financial relationships that could be construed as a potential conflict of interest.

## Publisher’s Note

All claims expressed in this article are solely those of the authors and do not necessarily represent those of their affiliated organizations, or those of the publisher, the editors and the reviewers. Any product that may be evaluated in this article, or claim that may be made by its manufacturer, is not guaranteed or endorsed by the publisher.
